# The expanded CAG repeat in the huntingtin gene as target for therapeutic RNA modulation throughout the HD mouse brain

**DOI:** 10.1371/journal.pone.0171127

**Published:** 2017-02-09

**Authors:** Nicole A. Datson, Anchel González-Barriga, Eleni Kourkouta, Rudie Weij, Jeroen van de Giessen, Susan Mulders, Outi Kontkanen, Taneli Heikkinen, Kimmo Lehtimäki, Judith C. T. van Deutekom

**Affiliations:** 1 BioMarin Nederland BV, Leiden, The Netherlands; 2 Charles River Discovery Research Services, Kuopio, Finland; University of Florida, UNITED STATES

## Abstract

The aim of these studies was to demonstrate the therapeutic capacity of an antisense oligonucleotide with the sequence (CUG)7 targeting the expanded CAG repeat in huntingtin (*HTT*) mRNA in vivo in the R6/2 N-terminal fragment and Q175 knock-in Huntington’s disease (HD) mouse models. In a first study, R6/2 mice received six weekly intracerebroventricular infusions with a low and high dose of (CUG)7 and were sacrificed 2 weeks later. A 15–60% reduction of both soluble and aggregated mutant HTT protein was observed in striatum, hippocampus and cortex of (CUG)7-treated mice. This correction at the molecular level resulted in an improvement of performance in multiple motor tasks, increased whole brain and cortical volume, reduced levels of the gliosis marker myo-inositol, increased levels of the neuronal integrity marker N-aceyl aspartate and increased mRNA levels of the striatal marker *Darpp-32*. These neuroanatomical and neurochemical changes, together with the improved motor performance, suggest that treatment with (CUG)7 ameliorates basal ganglia dysfunction. The HTT-lowering was confirmed by an independent study in Q175 mice using a similar (CUG)7 AON dosing regimen, further demonstrating a lasting reduction of mutant HTT protein in striatum, hippocampus and cortex for up to 18 weeks post last infusion along with an increase in motor activity. Based on these encouraging results, (CUG)7 may thus offer an interesting alternative HTT-lowering strategy for HD.

## Introduction

Huntington’s disease (HD) is a rare inherited neurodegenerative disorder with a progressive and fatal course characterized by movement disorders, cognitive impairment, dementia and psychiatric manifestations including depression and psychosis. These symptoms result from the selective death and dysfunction of specific neuronal subpopulations within the central nervous system, in particular of medium spiny GABAergic projection neurons in the striatum, although marked alterations have also been observed in other areas of the brain, including the cerebellar cortex, thalamus and cerebellum [1, 2]. Generally, first symptoms occur in midlife, eventually leading to premature death within 15–20 years after disease onset. The disease causing mutation is the expansion of a CAG-trinucleotide repeat in the coding region of exon 1 of the huntingtin (*HTT*) gene, which results in a mutant protein with an elongated polyglutamine (also known as polyQ) stretch at its N-terminus [3]. This expanded polyglutamine stretch confers a toxic gain-of-function to mutant protein forms, ultimately resulting in widespread neuronal death. Besides a toxic gain-of-function of mutant HTT (mHTT), loss of the normal functions of wildtype HTT (wtHTT) likely also plays a role in pathological mechanisms of HD [4]. In addition, there is emerging evidence that mRNA species with extended CAG repeats may themselves be directly involved in toxicity, possibly by sequestration of diverse proteins [5]. Today there is still a significant unmet need in disease-modifying therapies for the treatment and management of HD, with drugs that can alleviate some of the movement and psychiatric symptoms, but no curative treatments available [6].

Different “huntingtin-lowering” therapeutic strategies based on RNA modulation are in (pre-)clinical development, including adeno-associated virus (AAV)-assisted gene therapy approaches based on vectors expressing *HTT*-silencing siRNA or miRNA, zinc finger proteins (ZFPs) targeting the expanded CAG repeat or antisense oligonucleotides (AONs) to lower (mutant) HTT mRNA and/or protein levels (reviewed in [7]). These promising strategies are expected to slow down or prevent HD onset, since they act at the root cause of the disease, i.e. the toxicity of mutant huntingtin mRNA and protein molecules. The first randomized, placebo-controlled, dose escalation phase I/2a clinical study with a non allele-selective RNaseH-activating AON (IONIS-HTT_Rx_) has just recently started recruiting patients with early manifest HD (ClinicalTrials.gov Identifier: NCT02519036). While this is a first important step in the clinical development of HTT-lowering therapeutics, there is overall consensus that a more allele-selective suppression of the mHTT protein is likely a safer strategy, sparing the important and diverse cellular functions of the wtHTT protein, which includes a role in transcriptional regulation, intracellular transport of vesicles and organelles, energy metabolism, cell signalling, protein homeostasis, brain development and neuronal survival [8].

Two main allele-selective approaches are being explored, exploiting the presence of discriminative polymorphic regions in the wt*HTT* and m*HTT* mRNA. The first strategy makes use of either AONs with different chemistries or single stranded siRNAs (ss-siRNAs) that preferentially target m*HTT* transcripts with expanded CAG repeats [9–12]. A posed concern of this strategy is the potential for off-target effects on other CAG repeat containing transcripts [7]. The second strategy targets single nucleotide polymorphisms (SNPs) that segregate on a limited number of HD haplotypes, allowing design of AONs or siRNAs specifically targeting m*HTT* [13–15]. This strategy, however, requires the parallel development of multiple drugs each targeting a different SNP in m*HTT* and therefore serves only subsets of HD patients with a particular haplotype.

Animal models for HD play an important role in generating preclinical proof-of-concept (PoC) for abovementioned therapeutic strategies. The R6/2 mouse model is one of the first HD transgenic mouse lines produced and the most extensively studied and utilized mouse model of HD to date [16]. R6/2 is a transgenic N-terminal fragment model, expressing a relatively small 5’ part of the human *HTT* gene including exon 1 with 150 CAGs. R6/2 mice have a robust and rapidly developing phenotype with several HD-like characteristics and neuropathology, making them especially suitable for preclinical testing of therapeutic potential of compounds for HD. R6/2 mice recapitulate several of the neuroanatomical and neurochemical hallmarks observed in HD patients, including robust brain atrophy, a decrease in striatal *N*-acetyl aspartate (NAA) levels (a marker for neuronal health), a reduced expression of medium spiny neuron marker Ppp1r1b (also known as Darpp-32) and an increase of glial marker myo-inositol [17–19]. The Q175 HD mouse model is a knock-in model in which part of exon 1 and intron 1 of the endogenous mouse *Htt* gene was replaced by the corresponding *HTT* human segment with a repeat of around 179 CAGs [20, 21]. Knock-in models of HD carry the expanded CAG repeat within the native murine *Htt* gene and under the control of the endogenous mouse promoter, thus more closely recapitulate the genetic context of patients with HD than N-terminal fragment models. Q175 mice have robust, progressive and early-onset alterations in electrophysiological, morphological, volumetric and metabolic endpoints, with an overall milder phenotype in heterozygotes than homozygotes [20, 21].

Using a 2'-*O*-methyl phosphorothioate (2OMePS) (CUG)7 AON that specifically targets expanded CAG stretches and does not activate RNaseH knockdown, we have previously demonstrated lower detection of the mutant relative to the wt*HTT* transcript in HD patient-derived fibroblasts and lymphoblasts [9]. The aim of the current study was to confirm the therapeutic capacity of this (CUG)7 AON in vivo in the R6/2 HD and Q175 mouse models by investigating whether repeated intracerebroventricular (ICV) administration would not only result in HTT-lowering, but also improve several aspects of the HD-like phenotype. In both HD mouse models a significant reduction of mHTT protein was observed in multiple brain regions, which was associated with improved motor phenotype. Moreover, the HTT-lowering lasted for at least 18 weeks post last infusion.

## Results

### HTT-lowering in multiple key brain regions of (CUG)7 AON treated R6/2 mice

**Fig 1:**
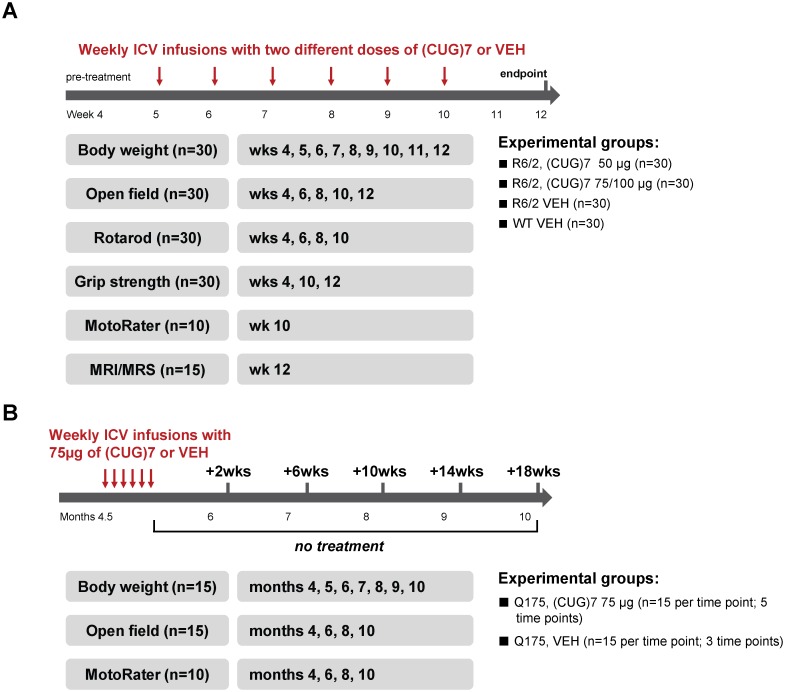
Study design in the HD mouse models. Study design in the R6/2 (A) and Q175 (B) HD mouse models, indicating dosing regimens, sample size per experimental group and time points at which the different neurobehavioral tests and imaging were performed.

To demonstrate therapeutic proof-of-concept (PoC) for the (CUG)7 AON an extensive R6/2 mouse study was performed using a large sample size (n = 30 per experimental group, both genders) and including several behavioral tests for motor function and MRI/MRS imaging (Fig 1). A total of 6 weekly ICV infusions (low or high dose (CUG)7 AON) were administered to the mice, starting at 5 weeks of age. HTT-lowering was investigated at both the mRNA and protein level.

We have previously reported on reduced detection of the *HTT* mRNA using Real time quantitative PCR (RT-qPCR) in HD patient-derived fibroblasts transfected with (CUG)7 [9]. Recent RNA cleanup data suggests that binding of (CUG)7 to the CAG repeat in the m*HTT* mRNA interferes with its RT-qPCR detection (S1 Fig). We detected a strong inhibition of RT-qPCR amplification of m*HTT* in RNA derived from cortex samples of (CUG)7-treated R6/2 mice with both (CUG)7 doses compared to vehicle (VEH) treatment (S1 Fig). No effect of (CUG)7 on RT-qPCR detection of the exon 1 of endogenous mouse *Htt* mRNA with only 4 CAGs was observed in both(CUG)7-treated R6/2 mice and VEH-treated controls (S1 Fig), indicating that the inhibition by (CUG)7 is dependent on CAG repeat length. Besides in cortex, RT-qPCR amplification of m*HTT* was also inhibited in striatum and hippocampus (S1 Fig), thalamus, olfactory bulb, cerebellum and brain stem (data not shown), suggesting that (CUG)7 distributes throughout the R6/2 mouse brain and is able to bind to m*HTT* transcripts in brain regions even remotely located from the site of infusion.

**Fig 2:**
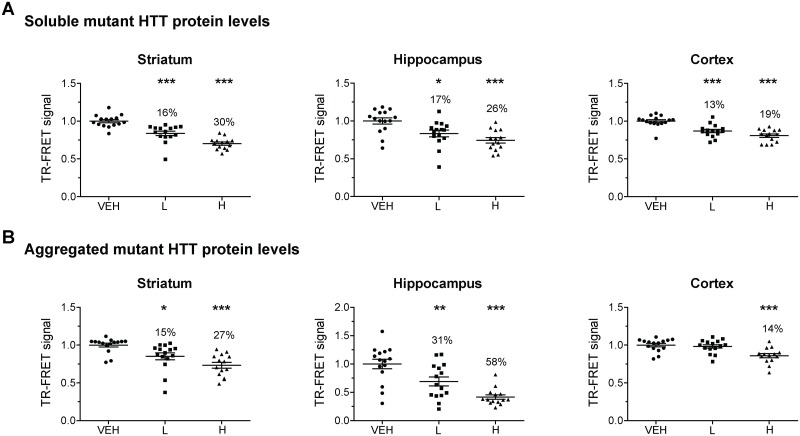
HTT-lowering analysis in different brain regions of R6/2 mice after a total of 6 ICV infusions (once weekly) with 2 different dosing regimens of (CUG)7 or VEH. Levels of soluble (A) and aggregated (B) mHTT protein, as determined by TR-FRET-based immunoassay [22]. The relative fluorescent signal obtained in the TR-FRET immunoassay is shown normlaised to levels in the VEH group (set at 1.0). Each point is the average of 3 technical replicates. Data are presented as mean ± SEM (n = 14–15). Significance was assessed using One Way ANOVA on R6/2 groups followed by Dunnett’s multiple comparison posthoc test (*p<0.05 **p<0.01, ***p<0.001 compared to R6/2 VEH).

Next we investigated the effect binding of (CUG)7 to the m*HTT* transcript had on mHTT protein levels. Mutant HTT protein levels were determined in brain tissue from striatum, hippocampus, cortex and cerebellum using the sensitive time-resolved Förster resonance energy transfer (TR-FRET) immuno assay which allows quantification of levels of both soluble and aggregated forms of mHTT [22]. A statistically significant and dose-dependent reduction of soluble mHTT protein levels was observed in striatum, hippocampus and cortex of R6/2 (n = 14–15) compared to VEH controls at both the low and high (CUG)7 dose level. Soluble mutant HTT protein levels were significantly reduced by 13–17% in the low dose and by 19–30% in the high dose treatment groups in all three brain regions (Fig 2A). Aggregated mHTT protein levels were also significantly reduced in a dose-dependent way, with a reduction of 15% in striatum and 31% in hippocampus in the low dose treatment group and of 27% in striatum, 58% in hippocampus and 14% in cortex in the high dose group (Fig 2B). In cerebellum the reduction of soluble mHTT levels was less pronounced and there was more variation in the levels of the aggregated form of the mHTT protein, but nevertheless resulted in a positive trend towards reduction was observed for both mHTT protein forms after (CUG)7 administration (soluble: p = 0.077, aggregated: p = 0.066) (S2B Fig). No differences in extent of soluble or aggregated mHTT protein reduction were observed between males and females.

### (CUG)7 AON improves motor performance of R6/2 mice

**Fig 3:**
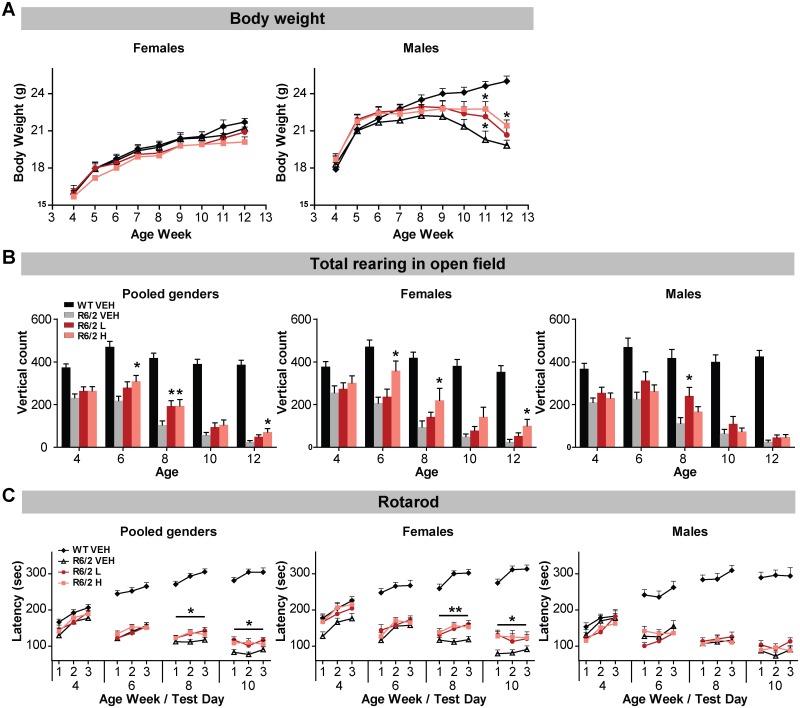
Effect of (CUG)7 treatment on motor performance of R6/2 mice. The effect of repeated ICV administration of (CUG)7 AON on body weight (A), total rearing frequency (B) and rotarod performance (C) of pooled genders (n = 22–31), female (n = 11–17)and male (n = 11–15) R6/2 mice from 4–12 weeks of age. (A) Body weight data over time are presented as mean ± SEM. Significance was assessed using One Way ANOVA on R6/2 groups followed by Dunnett’s multiple comparison posthoc test (*p < 0.05 compared to R6/2 VEH). A significant increase in body weight was observed in males at weeks 11 and 12 in the high dose group and at week 11 in the low dose group. (B) Vertical activity in the open field, also known as rearing, is represented as mean ± SEM. Significance was assessed using One Way ANOVA on R6/2 groups only followed by Dunnett’s multiple comparison posthoc test (*p<0.05 compared to R6/2 VEH). A significant increase in vertical count was observed in pooled genders at weeks 6, 8 and 12 in the high dose group and at week 8 in the low dose group, in females at weeks 6, 8 and 12 in the high dose group and in males at week 8 in the low dose group. (C) In the rotarod three consecutive accelerating trials were performed each week, indicated by 1, 2 and 3 and the latency to fall from the rod was recorded. Data are presented as mean ± SEM. Significance was assessed using One Way ANOVA on R6/2 groups followed by Dunnett’s multiple comparison posthoc test (*p < 0.05, **p<0.01 compared to R6/2 VEH).

Body weight was monitored throughout the study to assess safety and the potential therapeutic benefit of the (CUG)7 AON treatment. While body weight of R6/2 females in this study was not different than that of age-matched WT females, male R6/2 mice started to lose body weight compared to WT males starting around the age of 8–9 weeks, as anticipated [23]. A transient treatment-induced increase in body weight was however observed at 11 weeks in the male group treated with the high AON dose (p<0.05) (Fig 3A).

The therapeutic efficacy of repeated ICV administration of the (CUG)7 AON on motor performance was assessed in R6/2 mice using a battery of behavioral motor tests.

Open Field: Overall horizontal activity in the open field was not altered by treatment, as indicated by total distance traveled, distance traveled in the center area of the open field and average velocity (data not shown). However, a significant treatment effect was observed on vertical activity (total rearing frequency) with the higher dose of (CUG)7 at weeks 6, 8 and 12 of testing in pooled genders (Fig 3B). This treatment effect was mostly attributable to the females, in which the high dose group showed a significant increase in vertical count at weeks 6, 8 and 12 and a trend towards significance in week 10 (p = 0.053). Treatment did not affect the rearing frequency in the center area of the open field, although there was a trend at weeks 8 and 12 in pooled genders for the high (CUG)7 dose group (p-values 0.059 and 0.051 respectively, data not shown).

Rotarod: The effect of treatment on motor coordination and balance was assessed, as indicated by the latency to fall from the rod. A positive treatment effect was observed, primarily in female R6/2 mice treated with (CUG)7 at weeks 8 and 10 compared to VEH-treated controls (Fig 3C).

**Fig 4:**
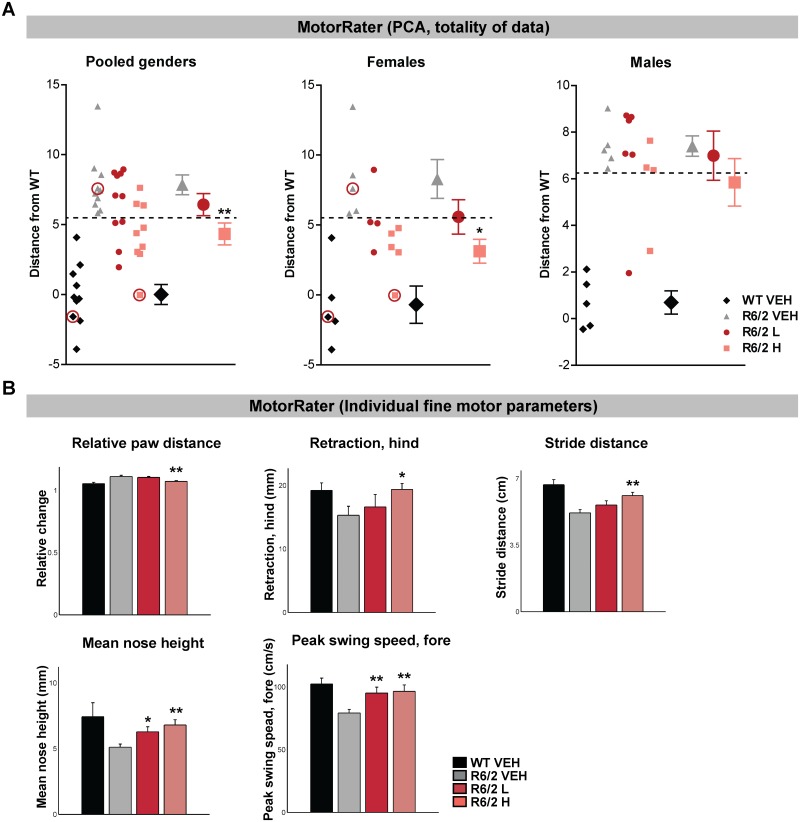
(CUG)7 effect on fine motor capabilities in R6/2 mice. (A) Principal component (PC) score of fine motor data as distance from average of WT. PC scores for each individual mouse were projected onto a line connecting the averages of WT and R6/2 groups within the PC space, and then, PC score distances from WT along that line were obtained. This analysis gives an overall score (based on 83 separate parameters) for individual mice (pooled genders (n = 10) and males and females separately (n = 5 per gender). Data of individual mice are presented (left side of each graph) in addition to the mean values ± SEM (right part of each graph). The dashed line indicates the lowest data point of the VEH-treated R6/2 mice. The 3 circled data points represent the mice shown in the videos in the supplementary material. Significance was assessed using One Way ANOVA on R6/2 groups followed by Dunnett’s multiple comparison posthoc test (*p<0.05, **p<0.01 compared to R6/2 VEH). (B) Example of 5 individual fine motor parameters significantly affected by treatment with (CUG)7 in pooled genders. Data are presented as mean ± SEM. Significance was assessed using One Way ANOVA on R6/2 groups followed by Dunnett’s multiple comparison posthoc test (*p<0.05, **p<0.01 compared to R6/2 VEH).

Fine Motor Capabilities and Gait: A clear improvement of fine motor performance was observed using 3D kinematic analysis of spontaneous movement. Principal component (PC) analysis of the fine motor data showed that the overall, combined PC score was significantly reduced in the high (CUG)7 dose treatment group in females, indicating that motor performance and gait was more similar to WTs after treatment (Fig 4A). This shift towards WTs was also evident in males in the high dose group but did not reach statistical significance. However, the effect in males did contribute to a lower p-value when data from pooled genders was analysed (p = 0.0073) compared to data in females alone (p = 0.014). A closer look into the pooled gender data revealed that principal component clusters 2, 6, and 7 contributed most to this shift towards WTs (S3A Fig). A total of 83 different parameters describing fine motor capabilities and details of gait were also analysed for pooled gender and males and females separately, including horizontal distance of front paw trajectory, retraction of the hind leg, stride distance, mean nose height and peak swing speed of the fore leg (pooled gender data in Fig 4B, data for females and males individually in S3 Fig). Overall, the majority of the significant fine motor changes observed in the (CUG)7-treated R6/2 mice shifted towards levels observed in WT controls and the improved movement and coordination was clearly apparent just by watching the videos of the mice walking in the fine motor assay (S1–S3 Videos). An overview of all fine motor parameters significantly altered by (CUG)7 treatment are listed in Table 1.

**Table 1:** Overview of (CUG)7 treatment effects observed on fine motor performance.

	ANOVA p-value	Posthoc p-value	
		(CUG)7 L	(CUG)7 H
**Fine Motor and Gait (Age Week 10):**			
- **Stride Speed**	p = 0.049 (f+m)	NS (f+m)	▲p = 0.028 (f+m)
- **Stride Distance**	p = 0.007 (f+m)	NS (f+m)	▲p = 0.004 (f+m)
- **Swing Peak Speed Fore**	p = 0.015 (f+m)	▲p = 0.032 (f+m)	▲p = 0.016 (f+m)
	p = 0.013 (f)	▲p = 0.032 (f)	▲p = 0.012 (f)
- **Swing Jerk Metric Hind**	p = 0.032 (m)	NS (m)	▲p = 0.026 (m)
- **Nose Height**	p = 0.006 (f+m)	NS (f+m)	▲p = 0.004 (f+m)
	p = 0.003 (f)	NS (f)	▲p = 0.003 (f)
	p = 0.015 (m)	NS (m)	▲p = 0.026 (m)
- **Paw Lift-off Angle Hind**	p = 0.001 (f+m)	▲p = 0.001 (f+m)	NS (f+m)
	p = 0.011 (f)	▲p = 0.027 (f)	▲p = 0.010 (f)
	p = 0.003 (f)	▲p = 0.006 (m)	NS (m)
- **Swing Traj Over Tresh 25 Hind**	p = 0.020 (f+m)	▲p = 0.038 (f+m)	NS (f+m)
- **Swing Traj Over Tresh 40 Hind**	p = 0.019 (f+m)	▲p = 0.024 (f+m)	NS (f+m)
- **Swing Traj Over Tresh 50 Hind**	p = 0.038 (f+m)	▲p = 0.030 (f+m)	NS (f+m)
	p = 0.019 (f)	▲p = 0.017 (f)	NS (f)
- **Swing Traj Over Tresh 60 Hind**	p = 0.019 (f)	▲p = 0.018 (f)	NS (f)
- **Horizontal Distance of Front Paw Trajectory**			
	p = 0.008 (f+m)	NS (f+m)	▲p = 0.007 (f+m)
- **PC3**	p = 0.042 (f)	NS (f)	▲p = 0.028 (f)
- **PC7**	p = 0.018 (f)	NS (f)	▲p = 0.012 (f)
- **Dist. from WT**	p = 0.028 (f+m)	NS (f+m)	▲p = 0.016 (f+m)
	p = 0.020 (f+m)	NS (f+m)	▲p = 0.011 (f+m)
	p = 0.026 (f)	NS (f)	▲p = 0.015 (f)

P-values of each analysis are indicated, for the one way ANOVA (all R6/2 groups) and posthoc testing (comparison to VEH). The arrows indicate the change of the parameter towards WT levels. NS: not significant. m: males; f: females.

Grip strength: No significant effects of treatment with (CUG)7 were observed on grip strength (data not shown).

In summary, a positive and significant effect of ICV treatment with the (CUG)7 AON was observed in several of the motor tests performed, including the open field, rotarod and fine motor kinematic assay.

### (CUG)7 AON increases whole brain and cortical volume in the R6/2 mouse

**Fig 5:**
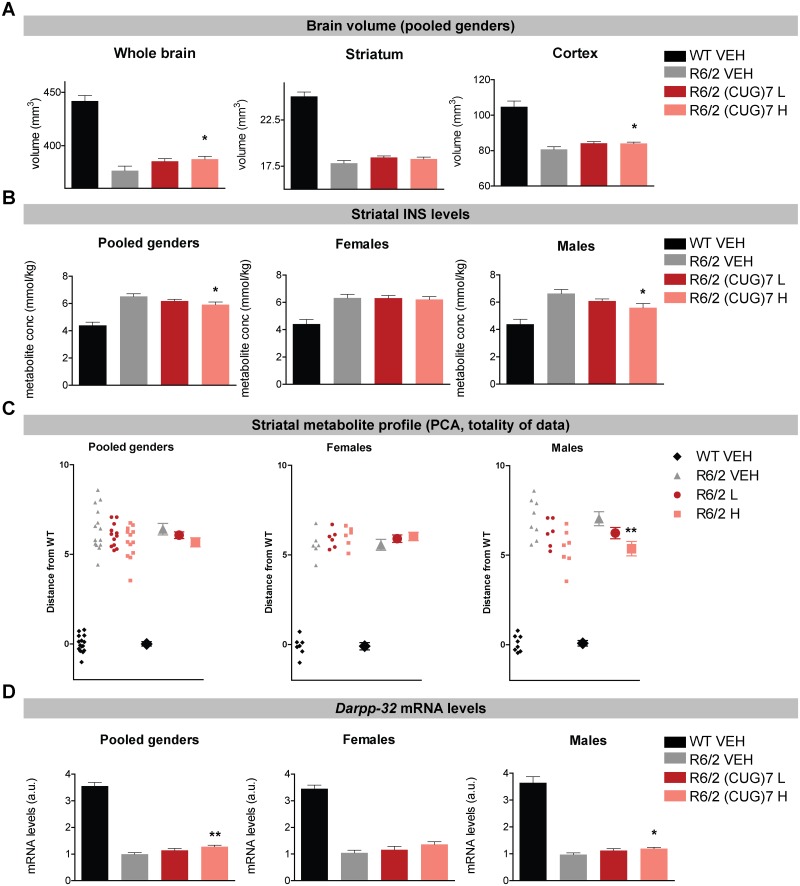
Effects of (CUG)7 on brain volume and neurochemical profile of striatum of R6/2 mice. (A) MRI analysis of whole brain, cortical and striatal volume in (CUG)7-treated and VEH-treated R6/2 mice at 12 weeks of age in pooled genders (n = 12–15). Data are presented as mean ± SEM. ANOVA p-values to assess differences between groups were just above the p-value threshold of 0.05 in cortex and whole brain in pooled genders (cortex: p = 0.051; whole brain: p = 0.063). Using a 2-tailed t-test a significant increase in whole brain and cortical volume was observed for the high (CUG)7 dose groups compared to R6/2 VEH (*p<0.05) and a trend for the low dose group in cortex (p = 0.058). Results for females (n = 6–7) and males (n = 7–9) were not significant (data not shown). (B) The effect of (CUG)7-treatment on the concentrations of striatal metabolite myo-inositol (INS) in pooled genders (n = 12–15), female (n = 6–7) and male (n = 7–9) R6/2 mice at 12 weeks of age as measured by in vivo MR spectroscopy. Data are presented as mean ± SEM. ANOVA p-values to assess differences between groups were significant for males (p = 0.035) and displayed a trend for pooled genders (p = 0.070). Using a 2-tailed t-test a significant decrease in striatal INS levels were observed in the high dose treatment group in males and pooled genders (*p<0.05 compared to R6/2 VEH). (C) Principal component (PC) score of MRS data as distance from average of WT. PC scores for each individual mouse were projected onto a line connecting the averages of WT and R6/2 groups within the PC space, and then, PC score distances from WT along that line were obtained. This analysis gives an overall score (based on 16 separate metabolites) for individual mice (pooled genders: n = 12–15; females: n = 6–7 and males: n = 7–9). Significance was assessed using One Way ANOVA on R6/2 groups followed by Dunnett’s multiple comparison posthoc test (**p<0.01 compared to VEH). A significant shift towards WTs was observed in males in the high dose treatment group. (D) *Darpp-32* mRNA levels in striatum determined by RT-qPCR in pooled genders (n = 12–15), females (n = 6–7) and males (n = 7–9). *Darpp-32* mRNA levels are expressed relative to levels after VEH-treatment and normalized against averaged expression levels of *Ywhaz*, *Rab2* and *Gapdh*. Data are presented as mean ± SEM. Using a 2-tailed t-test a significant increase in striatal *Darpp-32* mRNA levels were observed in the high dose treatment group in pooled genders and males (*p < 0.05, **p<0.01 compared to R6/2 VEH). In females a similar trend was observed in the high dose group (p = 0.053).

At the age of 12 weeks (two weeks after the last ICV infusion) the R6/2 mice were subjected to in vivo T2-MRI analysis to quantify brain volume. Using a 2-tailed t-test a significant increase in whole brain and cortical volume was observed in pooled genders for the high (CUG)7 dose groups compared to R6/2 VEH and a trend for the low dose group in cortex (p = 0.058) (Fig 5A). Differences between individual genders were not significant (data not shown).

### (CUG)7 AON improves neurochemical hallmarks of HD in the striatum of R6/2 mice

R6/2 mice were also subjected to 1H-MR Spectroscopy (MRS) analysis of striatal metabolites. Using a 2-tailed t-test a significant decrease in striatal levels of the glial marker myo-inositol (INS) was observed in males and pooled genders treated with the high dose of (CUG)7 compared to vehicle-treated R6/2 (Fig 5B). No significant effects were observed in females. Furthermore, in males a significant increase of levels of the neuronal health marker N-acetyl aspartate (NAA) was observed in the low dose treatment group using a 2-tailed t-test (p = 0.045; data not shown). Both INS and NAA are striatal metabolites that have been consistently shown to display altered levels in the striatum of HD animal models and patients. While several other striatal metabolites, such as creatine, phosphocreatine and glutamate, showed a shift in the direction of the WTs in particular in males, significance was not reached (data not shown). This was reflected in the principal component (PC) analysis of the MRS data, which showed that the overall, combined PC score was significantly reduced in the high (CUG)7 dose treatment group in males, indicating that the overall striatal metabolite profile was more similar to WTs after treatment (Fig 5C).

In parallel with the improved striatal INS metabolite profile, a small but significant increase in mRNA levels of the striatal marker *Darpp-32* was observed with the highest (CUG)7 dose (Fig 5D) in pooled genders and in males. In females the increase in *Darpp-32* expression in the high dose group was just above the p-value cut off of 0.05 (p = 0.053). *Darpp-32* expression is lower in the striatum of HD animal models and patients, in line with the widespread loss of medium spiny neurons in this brain region.

In summary, small but significant changes in levels of INS, NAA and *Darpp-32* were observed in (CUG)7-treated animals, changing in the direction that would be expected in case of a therapeutic benefit, i.e. towards levels observed in wildtype mice.

### (CUG)7-mediated mHTT protein lowering in multiple brain regions is replicated in the Q175 HD mouse model and lasts for at least 18 weeks post infusion

To confirm the (CUG)7-induced HTT-lowering observed in R6/2 in a second HD mouse model and assess how long the HTT-lowering persisted, Q175 knock-in mice were treated with the same number of ICV infusions of a 75 μg dose of (CUG)7 (Fig 1B). In addition to the 2 weeks post last infusion time point analysed in the R6/2 study, 4 additional time points were added using separate cohorts of mice: 6, 10, 14 and 18 weeks post last infusion.

**Fig 6:**
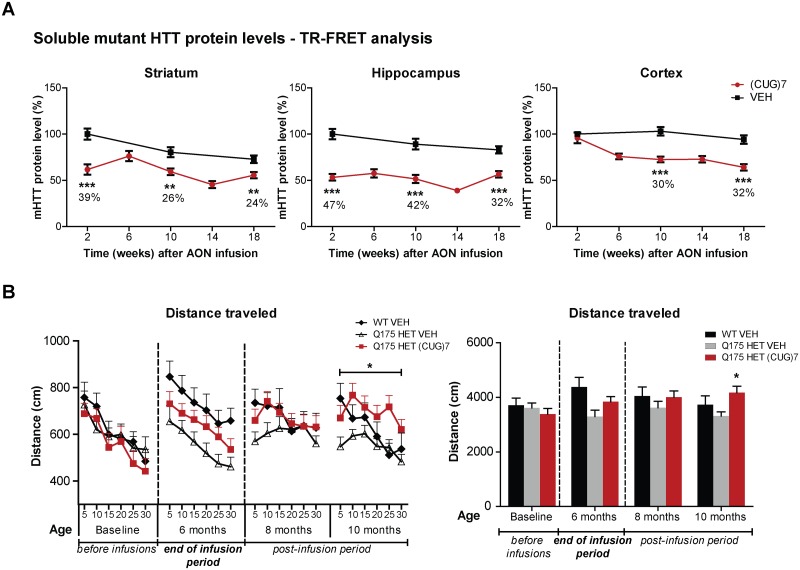
Effects of (CUG)7 treatment in the Q175 HD mouse model. (A) Levels of soluble mHTT protein in striatum, hippocampus and cortex, as determined by TR-FRET-based immunoassay [22]. Each point is the average of 3 technical replicates. Soluble mHTT protein levels are expressed relative to levels after VEH-treatment at the 2 week post infusion time point (set to 100%). Data are presented as mean ± SEM. Significance was assessed using a 2-tailed t-test comparing (CUG)7-treated mice to VEH controls per time point (**p<0.01, ***p<0.001). (B) Distance traveled in the open field at baseline and different time points post infusion. Mice were placed in the center of the chamber and their behavior was recorded for 30 min in 5-minute bins (individual bins shown in left graph, cumulative distance in right graph). A significant increase in distance traveled was observed in 10-month old (CUG)7-treated Q175 mice, which corresponds to 4 months post last infusion (around the 18 week post infusion time point). Data are presented as mean ± SEM. Significance was assessed using a 2-tailed t-test comparing (CUG)7- treated Q175 mice to VEH controls per time point (*p<0.05).

Using TR-FRET, a significant reduction of soluble mutant HTT protein levels was observed in Q175 cortex from 6 weeks post infusion onwards up to the last time point of 18 weeks post infusion (Fig 6A). Remarkedly, over time a reduction of mHTT protein levels was observed in striatum and hippocampus of VEH-treated mice, which may reflect temporal changes in mHTT expression level in these brain regions. To take this change in mHTT levels into account, the reduction in (CUG)7-treated mice was expressed as a percentage of VEH levels per VEH-controlled time point. In striatum and hippocampus soluble mHTT protein levels were already significantly reduced at 2 weeks post infusion, also lasting for up to 18 weeks post last infusion. The magnitude of the protein reduction ranged from 24–47%, depending on the brain region and time point (Fig 6A). Similar to the R6/2 study, the protein reduction was largest in hippocampus.

HTT-lowering was accompanied by an increase in motor activity, with an increased distance travelled in the open field at the age of 10 months (corresponding to 4 months post infusion), even bypassing WT controls (Fig 6B). The mild phenotype of the Q175 heterozygotes up to 10 months of age did not generate a sufficient window to detect changes in fine motor performance and gait using the MotoRater.

### (CUG)7 does not affect protein levels of endogenous mouse genes with CAG repeats

**Fig 7:**
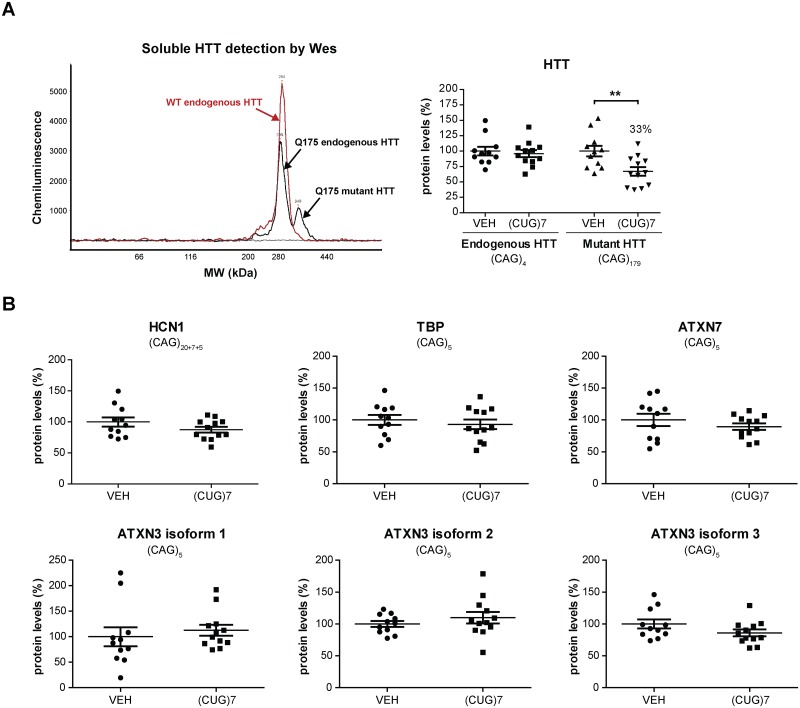
(CUG)7 effect on protein levels of mouse genes with non-expanded CAG-repeats. (A) Levels of soluble mHTT protein quantified in Q175 cortex 18 weeks post last infusion using Wes analysis. The left graph depicts the chemiluminescence profile obtained using an antibody recognizing an epitope in exon 15 of HTT. A typical plot obtained in cortex of Q175 mice (in black) and in WT littermates (in red) is depicted. Only a single peak is present in WT mice representing mouse endogenous HTT protein, while in Q175 knock-in mice an additional peak with a higher molecular weight (MW) is visible representing mHTT protein with the expanded CAG repeat. In the right graph the data obtained with this antibody is shown. Data are presented as mean ± SEM. Significance was assessed for both endogenous and mutant HTT protein using a 2-tailed t-test comparing (CUG)7-treated Q175 mice to VEH controls (**p<0.01). (B) Protein levels of HCN1 (Hyperpolarization Activated Cyclic Nucleotide Gated Potassium Channel 1), TBP (TATA-Box Binding Protein), 3 different isoforms of ATXN3 (Ataxin 3) and ATXN7 (Ataxin 7) quantified in Q175 cortex 18 weeks post last infusion using Wes analysis. The number of uninterrupted CAGs is indicated in each graph. Data are presented as mean ± SEM. Significance was assessed using a 2-tailed t-test comparing (CUG)7-treated Q175 mice to VEH controls, but no significant differences were observed for any of the proteins tested.

After having demonstrated that the (CUG)7 AON also reduces levels of mutant HTT protein throughout the Q175 mouse brain, we next evaluated its effect on protein levels of several endogenous mouse genes with shorter non-expanded CAG repeat stretches, including the Q175 mouse endogenous HTT. Using a second independent method, Wes analysis, the mHTT protein reduction observed in cortex at the 18 week post infusion time point was replicated and in line with the TR-FRET data a 33% reduction of mHTT protein was observed in (CUG)7-treated compared to VEH-treated Q175 mice using an antibody raised against the region encoded by exon 15 of HTT (Fig 7A). No effect of (CUG)7 on mouse endogenous HTT protein was observed (Fig 7A). In addition, protein levels of HCN1 (20 + 7 + 5 uninterrupted CAGs), TBP (5 CAGs), ATXN7 (5 CAGs) and ATXN3 (5 CAGs) were analysed and also no effect of (CUG)7 was observed on expression levels of any of these proteins (Fig 7B). For ATXN3 several alternatively spliced transcript variants have been described, giving rise to different ATXN3 isoforms [24]. We detected 3 distinct ATXN3 isoforms using Wes analysis, none of which showed any reduction by (CUG)7 (Fig 7B).

## Discussion

The aim of these studies was to demonstrate the therapeutic capacity of a 2OMePS RNA (CUG)7 AON that does not activate RNase H and targets the expanded CAG repeat sequence in exon 1 of m*HTT*. In 2 different HD mouse models, the R6/2 N-terminal fragment model and the Q175 knock-in model, only 6 repeated weekly ICV administrations of (CUG)7 already reduced levels of the mHTT protein up by approximately 15–60% in 3 key brain regions affected in HD, i.e. striatum, cortex and hippocampus. This level of mHTT-lowering resulted in correction of the motor phenotype in multiple motor tests, a brain volume increase, positive changes in striatal metabolite profile and an increase of the striatal marker *Darpp-32*. The Q175 study further demonstrated that the HTT-lowering was persistent and lasted for up to 18 weeks post infusion, which suggests that a less frequent dose regimen is feasible.

**Fig 8:**
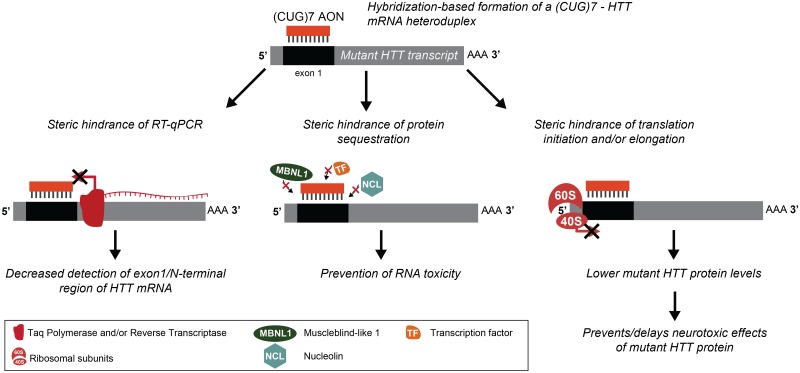
Proposed mechanism of action of (CUG)7. Binding of (CUG)7 to m*HTT* mRNA causes steric hindrance of RT-qPCR, which results in reduced detection of the transcript at the exon1 region. In addition, the (CUG)7-m*HTT* mRNA heteroduplex sterically hinders translation of the mutant protein, leading to lower expression levels. Moreover, binding of (CUG)7 may also play a role in reducing RNA neurotoxicity of m*HTT* by preventing sequestration of important cellular proteins such as muscleblind-like 1 (MBNL1), nucleolin (NCL) and diverse transcription factors (TF).

Our current hypothesis for the mechanism of action of the (CUG)7 AON is that its binding to the m*HTT* transcript not only results in a steric hindrance of RT-qPCR of the exon 1 but also sterically hinders translation initiation and/or elongation, resulting in lower mHTT protein levels (Fig 8). This interference with RT-qPCR detection of m*HTT* can be considered a proxy for (CUG)7 binding, and is abolished by a stringent cleanup procedure of the RNA involving a denaturation step to remove bound AON (S1 Fig). Interestingly, another group recently reported a similar observation using a locked nucleic acid (LNA)-modified CAG-targeting AON [25]. In striatum of R6/2 mice treated with the LNA-modified AON RT-qPCR amplification across the CAG repeat of HTT exon 1 was blocked, while this was not the case for an amplicon downstream of the CAG repeat [25].

In contrast to the study by Rué et al. [25], where no effect on mHTT protein was observed, in both the R6/2 and Q175 studies described herein, mHTT protein levels were signficantly reduced, suggesting that (CUG)7 bound to m*HTT* mRNA indeed interferes with protein synthesis. Consistent with our hypothesis, steric hindrance of protein synthesis has also been proposed to play a role in other CAG-targeting HTT-lowering approaches [10, 12].

Besides hindering protein synthesis, binding of (CUG)7 may also play a role in reducing RNA neurotoxicity of m*HTT* by preventing sequestration of important cellular proteins such as muscleblind-like 1 (MBNL1), nucleolin (NCL) and diverse transcription factors and/or preventing RAN-translation [5] (Fig 8). While we currently do not have any data to support this, it is intriguing that Rué et al. observed an improvement of the HD-like phenotype of R6/2 after intrastriatal injection of a CAG-targeting LNA-modified AON, without reduction of HTT protein levels, suggesting that reversal of the detrimental effects of m*HTT* RNA toxicity may play an important yet so far underappreciated role in HD pathogenesis [25].

We have previously observed a differential effect of (CUG)7 inhibition of RT-qPCR of the non-expanded WT versus expanded mutant *HTT* exon 1 region [9]. In our current line of thinking the higher (CUG)7 binding potential of expanded CAG repeats would not only result in a stronger inhibition of RT-qPCR of this region, but also in a stronger steric hindrance of mHTT protein synthesis, providing a basis for the allele-selectivity of the AON. We further investigated whether (CUG)7 had any effect on RT-qPCR detection of the endogenous mouse *Htt* gene. Since mouse *Htt* has a very short stretch of only 4 uninterrupted CAGs, we did not consider it likely that binding of (CUG)7 to this mRNA would occur due to a too low target affinity. Consistent with this expectation, no inhibitory effect of (CUG)7 was observed on RT-qPCR detection of mouse *Htt* in cortex in both R6/2 dose groups (S1A Fig). Furthermore, no effect on endogenous mouse HTT protein levels was observed in Q175 cortex 18 weeks post last infusion, while mHTT protein levels were reduced by 33% at this time point (Fig 7A). We expanded this data by also quantifying protein levels of 4 additional mouse genes with non-expanded CAG repeats, ranging from 5 to over 20 CAGs, and did not observe any reduction of protein levels of these genes (Fig 7B). While these data are encouraging and suggest limited off-target effects of (CUG)7 on non-expanded CAG repeats, this requires further investigation.

The reduction of mHTT protein levels obtained in the R6/2 brain resulted in a clear improvement of motor performance, as demonstrated by fine motor kinematic analysis in the MotoRater, with multiple parameters showing a signficant shift towards WTs. This relatively new behavioural tool allows 3D natural movement to be assessed in a fully automated, standardized quantitative and objective manner, providing readouts of movements of all relevant body parts, i.e. forelimbs, hindlimbs, trunk and tail. This results in a complete profile of the animal's motor abilities that extends far beyond mere foot prints. The MotoRater results were corroborated by a positive effect on latency on the rotoarod and an increase in rearing frequency.

The reduction of mHTT protein was also accompanied by an improvement of the neurochemical profile characteristic of HD in the striatum of R6/2 mice treated with the (CUG)7 AON. R6/2 mice display higher levels of the striatal metabolite and gliosis marker myo-inositol (INS) and lower levels of N-acetyl aspartate (NAA), a marker of neuronal integrity, consistent with changes that have been observed in the putamen of HD patients. In a recent longitudinal study of in vivo brain metabolite profiles in HD over a 24 month period, NAA was lower in pre-manifest and early HD than in controls whereas INS was robustly elevated in early HD [26]. Furthermore, total NAA levels inversely correlated with disease burden score, suggesting that this metabolite may be useful in identifying neurochemical responses to therapeutic agents. Interestingly, we observed a significant decrease in INS in the striatum of R6/2 mice upon treatment with the (CUG)7 AON, in addition to an increase in NAA, with both metabolites shifting in the direction that would be expected of a therapeutic benefit.

Another well-established neurochemical correlate of HD is the reduction of Darpp-32 expression in the striatum of HD mouse models [16, 27, 28]. Darpp-32 plays a key role in integrating neurotransmitter and neuromodulator signals in the GABAergic medium-size spiny neurons in the striatum, in particular in response to dopamine and glutamate and therefore upregulating its expression may alleviate disturbances in dopamine transmission that contribute to motor deficits in HD mice [29]. We indeed observed an increase in *Darpp-32* mRNA levels in the striatum of R6/2 mice after treatment with (CUG)7, which together with the changes in striatal metabolites (i.e. INS and NAA) and the improved motor performance suggests that treatment with (CUG)7 leads to an improvement of basal ganglia dysfunction.

Besides the improved striatal metabolite profile, the data also demonstrated an increase of whole brain and cortical volume in the (CUG)7-treated R6/2 mice. Changes in brain volume in R6/2 mice start to occur relatively early after onset of first symtoms, and are in first instance characterised by a reduction of growth of the brain followed by a loss of striatal, cortical, thalamic, hypothalamic and overall anterior brain volume [30]. The increase in cortical and whole brain volume we observed in treated R6/2 mice points towards a beneficial effect of (CUG)7, which counteracts the reduction of growth and loss of brain volume observed in R6/2.

The overall effect on functional and neurochemical parameters was more pronounced in the high dose treatment group and correlated with the stronger reduction of mHTT protein observed in this group compared to the low dose treatment group.

Gender differences in disease severity and progression in HD have been reported, with female HD patients having a slightly more severe phenotype and faster rate of progression in especially the motor and functional domains [31, 32]. Sex hormones may underlie these observed differences in HD [32, 33]. Gender differences have also been observed in several animal models for HD, affecting motor performance and survival, neuropathology and response to therapeutic intervention [34–36]. It is therefore recommended that both genders are included in preclinical studies testing new HD therapeutics in HD mouse models [37]. In the R6/2 study reported herein, mice of both genders were used and relatively large experimental groups (n = 15 per gender) to allow sufficient statistical power to detect effects in individual genders. Similar to previous findings, we also observed that several of the (CUG)7 treatment effects observed in R6/2 mice were gender-specific or more pronounced in one of both genders, such as for example the positive treatment effect on bodyweight and striatal neurochemical profile (INS/NAA/*Darpp-32*) in males and the improved motor performance in the rotarod and MotoRater in females. Female R6/2 mice have a somewhat slower disease progression and less severe motor phenotype than male R6/2 mice [23, 38], which may be easier to correct or may become evident sooner than in males.

Since (CUG)7 targets expanded CAG repeats it potentially may offer a more widespread therapy for other polyglutamine disorders besides HD, such as several spinocerebellar ataxias (SCA1, SCA2, SCA3, SCA17), dentatorubral-pallidoluysian atrophy (DRPLA) and spinal-bulbar muscular atrophy (SBMA). We have previously demonstrated reduced detection of *ATXN1*, *ATXN3* and *ATN1* mRNA transcripts with expanded CAG-repeats in cell lines from patients with SCA1, SCA3 and DRPLA respectively, indicating (CUG)7 binding to the expanded CAG stretches in these genes [9]. Since we hypothesize that (CUG)7 interferes with translation initiation and/or elongation, we consider it likely that steric hindrance of protein synthesis by (CUG)7 will also occur in these polyQ diseases. Indeed, repression of ATXN3 protein expression has been reported using peptide nucleic acid (PNA)-peptide conjugates targeting the expanded CAG repeat in exon 10 of *ATXN3* mRNA [11]. While additional in vivo PoC studies still need to be performed for these diseases in specific disease animal models, the clinical development trajectory could be significantly shorter than if different compounds need to be developed for each specific disorder, which holds promise given the high unmet medical need for several of these diseases and is an advantage compared to other HTT-lowering AONs in development for HD.

## Materials and methods

### CAG-repeat targeting antisense oligonucleotide

The CAG-repeat targeting antisense oligonucleotide used in all experiments was a 2OMePS modified oligoribonucleotide (21-mer) with the sequence 5'-C*UGC*UGC*UGC*UGC*UGC*UGC*UG-3’ (abbreviated as (CUG)7) in which all cytosine residues were 5-methyl-C modified (C* = 5-methylcytosine; U = uracil, G = guanine). (CUG)7 was developed to selectively target a pure CAG repeat sequence, such as the sequence present in exon 1 of the *HTT* transcript. AONs were synthesized by BioMarin Nederland B.V. (Leiden, The Netherlands).

### Experimental design of in vivo studies in R6/2 mice

R6/2 mice and wildtype (WT) littermate control mice (F_1_ generation) were bred at Charles River by mating (F_0_ generation) WT males (C57BL/6J, JAX, stock 000664) with ovarian transferred transgenic females (JAX, stock 006494). Mice were genotyped by PCR. All animal experiments were carried out according to Health guidelines for the care and use of laboratory animals (NIH), and approved by the National Animal Experiment Board of Finland. (CUG)7 AON was formulated in artificial CSF (aCSF; Harvard Apparatus, Massachusetts, USA).

An extensive PoC study was performed in R6/2 mice and WT controls. In setting up groups for study (i.e. vehicle or compound treated), mice were randomized into groups so that whole litters of mice did not end up in a single testing group. Because gender differences in numerous endpoints and in response to therapeutic interventions have been observed in preclinical studies, mice of both sexes were included in relatively large numbers (n = 15 per gender per experimental group) with sufficient statistical power to allow detection of potential gender specific effects, as recommended [37]. Mice were assigned to the following experimental groups: (1) R6/2 vehicle (VEH); (2) R6/2 AON low dose (50 μg); (3) R6/2 AON high dose (100 μg); (4) WT vehicle (VEH). At 5 weeks of age mice were implanted unilaterally with a cannula into the right lateral ventricle to allow for intracerebroventricular (ICV) infusion of AON or VEH (aCSF). Infusions (5 μl per infusion at a rate 0.3 μl/min) started at 5 weeks of age and continued once weekly for 6 weeks until the mice were 10 weeks of age. Two weeks post last infusion at the age of 12 weeks the mice were sacrificed and different brain regions were collected for RT-qPCR of mutant *HTT* mRNA (ipsilateral hemisphere) and TR-FRET analysis and soluble and aggregated mHTT protein (contralateral hemisphere) expression levels. Throughout the study bodyweight was monitored twice weekly. Unexpectedly, we observed an increase in seizure activity directly following administration of the high (CUG)7 dose in these R6/2 mice of 5 weeks of age. Therefore it was decided to lower the dose in the 100 μg group to 75 μg for the remaining infusions. R6/2 mice are known to have an increased susceptibility to develop seizures [16, 39, 40]. Despite the seizures, bodyweight was normal and locomotion and motor performance were not adversely affected but rather improved compared to VEH-treated R6/2 mice (further discussed in results section).

During the study the mice were subjected to a battery of behavioural tests for motor performance, as indicated in Fig 1. At 11 weeks of age the cannulas were removed under anesthesia and 15 mice per group (both genders) were subjected to Magnetic Resonance Imaging (MRI) to quantify brain volume and Magnetic Resonance Spectroscopy (MRS) to quantify levels of striatal metabolites. More details of the behavioural tests and the MRI/MRS are given below. At 12 weeks of age the mice were euthanized by an overdose of CO_2_ and subjected to tissue sampling for molecular analyses of HTT mRNA and protein. Motor function of the mice was assessed in several behavioural tests. Prior to testing, the mice were allowed to acclimatise to the experimental room conditions for at least 1 hour. Baseline assessments at 4 weeks were performed for rotarod, open field and grip strength followed by additional tests at specific time points (Fig 1). At 10 weeks fine motor behaviour of 10 mice/group was assessed using a high precision kinematic analysis.

Open Field Test: Activity chambers (Med Associates Inc, St Albans, VT; 27 x 27 x 20.3 cm) were equipped with infrared beams. Mice (n = 30 per experimental group) were placed in the center of the chamber and their behavior was recorded for 30 min in 5-minute bins. Quantitative analysis was performed on the following five dependent measures: total locomotion, locomotion in the center of the open field, rearing rate in the center, total rearing frequency and velocity. Animals were tested at low-stress conditions with the lighting lowered to approximately 10–30 lux of red light.

Rotarod: Each session included a training trial of 5 min at 4 RPM on the rotarod apparatus (AccuScan Instruments, Columbus, USA). One hour later, the animals (n = 30 per experimental group) were tested for 3 consecutive accelerating trials of 6 min with the speed changing from 0 to 40 RPM over 360 seconds and an inter-trial interval at least 30 min. The latency to fall from the rod was recorded. Mice remaining on the rod for more than 360 s were removed and their time scored as 360 sec.

Grip strength: One at a time, the mice (n = 30 per experimental group) were placed on the grip strength apparatus (San Diego Instruments, San Diego, USA) in such a way that the animal grabbed a small mesh grip with its forepaws. The entire apparatus was placed on a table top for testing. Animals were lowered to the platform and then slowly pulled away from the handle by the tail until the animal released the handle.

Fine Motor and Gait Analysis: Fine motor skills and gait properties were assessed (n = 10 per experimental group) at the age of 10 weeks using a high precision kinematic analysis method (MotoRater, TSE Systems, Homburg, Germany) using the walking mode. This relatively new motor test allows analysis of general gait pattern parameters, body posture, balance and fine motor skills in a fully automated setup. Before the test sessions the mice were marked in appropriate points of body, such as joints of limbs and parts of tail to ease the data analysis process. The movement data were captured using a high speed camera (300 frames / second) from three different dimensions, from below and both sides. The captured videos of each mouse were first converted to SimiMotion software to track the marked points of body to have the raw data i.e. the movement of the different body points in coordinates in relation to the ground, and each of the three dimensions was correlated. Different gait patterns and movements, observed to be genotype specific in R6/2 mice were analyzed using a custom made automated analysis system. The analyzed parameters included e.g.: 1) general gait pattern parameters (stride time and speed, step width, stance and swing time during a stride, interlimb coordination), 2) body posture and balance (toe clearance, iliac crest and hip height, hind limb protraction and retraction, tail position and movement), and 3) fine motor skills (swing speed during a stride, jerk metric during swing phase, angle ranges and deviations of different joints, vertical and horizontal head movement). The analysis provided altogether 83 different parameters related to fine motor capabilities and gait. Data were analyzed for distinctive parameters, as well as using principal component (PC) analysis for the acquired parameters.

### MRI and MRS

MRI acquisitions were performed (n = 15 per experimental group) at the age of 12 weeks using a horizontal 11.7T magnet with a bore size of 160 mm, equipped with a gradient set capable of max. gradient strength of 750 mT/m and interfaced to a Bruker Avance III console (Bruker Biospin GmbH, Ettlingen, Germany). A volume coil (Bruker Biospin GmbH, Ettlingen, Germany) was used for transmission and a surface phased array coil for receiving (Rapid Biomedical GmbH, Rimpar, Germany). Mice were anesthetized using isoflurane, fixed to a head holder and positioned in the magnet bore in a standard orientation relative to gradient coils. Anatomical images were acquired using a TurboRARE sequence with TR/TE = 2500/36ms, matrix size 256x256, FOV 20.0x20.0 mm^2^, 19 contiguous 0.7 mm thick slices and 8 averages.

1H-MRS data were collected using the same experimental setup. Voxel of 1.8x1.8x2.0 mm^3^ was placed in the striatum of the mouse based on T2-weighted images collected as described above. Automatic FASTMAP shimming algorithm was used to adjust B0 homogeneity in the voxel. The water signal was suppressed using variable power RF pulses with optimized relaxation delays (VAPOR) to obtain B1 and T1 insensitivity. A PRESS sequence (TE = 10 ms) combined with outer volume suppression (OVS) was used for the pre-localization. Three OVS blocks were used interleaved with water suppression pulses. Data were collected by averaging 512 excitations (frequency corrected for each FID) with TR of 2 s, number of points 4096 and spectral width of 5 kHz. Excitation frequency was shifted -2 ppm, to minimize the chemical shift phenomenon within the selected voxel. In addition, a reference spectrum (NT = 8) without water suppression was collected from the identical voxel using the same acquisition parameters. Peak areas for metabolites were analyzed using LCModel (Stephen Provencher Inc., Oakville, Canada) and results are given relative to water content in the tissue.

### Experimental design of in vivo time course study in Q175 knock-in mice

Male Q175 heterozygote mice and wildtype (WT) littermate control mice (F_1_ generation) were bred at Charles River. Mice were genotyped by PCR. All animal experiments were carried out according to Health guidelines for the care and use of laboratory animals (NIH), and approved by the National Animal Experiment Board of Finland. (CUG)7 AON was formulated in artificial CSF (aCSF; Harvard Apparatus, Massachusetts, USA).

Due to the large numbers of mice required for this study (a total of 120), it was decided to only include a single gender (males) in this study. In setting up groups for the time course study (i.e. vehicle or compound treated), mice were randomized into groups so that whole litters of mice did not end up in a single testing group. Mice (n = 15 per experimental group per time point, males only) were assigned to the following experimental groups: (1) Q175 AON (75 μg) (5 cohorts of n = 15 mice each sacrificed at 2, 6, 10, 14 and 18 weeks post last infusion) (2) Q175 vehicle (VEH) (3 cohorts of 15 mice each sacrificed at 2, 10 and 18 weeks post last infusion). At 4.5 months of age mice were implanted unilaterally with a cannula into the right lateral ventricle to allow for intracerebroventricular (ICV) infusion of AON or VEH (aCSF). Infusions (5 μl per infusion at a rate 0.3 μl/min) started at 4.5 months of age and continued once weekly for 6 weeks until the mice were 6 months old. During the study bodyweight was monitored twice weekly and the mice were subjected to 2 different behavioural tests for motor performance (Open field and MotoRater; performed in the same way as for R6/2), as indicated in Fig 1. Cohorts of mice were sacrificed at different times post last infusion by an overdose of CO_2_ and different brain regions were collected for TR-FRET analysis of soluble mHTT protein expression levels.

### Tissue processing

Mouse brains were collected directly upon sacrifice and different brain regions were dissected from the left and right hemisphere separately. All collected tissues were snap frozen in isopentane au bain marie in ethanol on dry ice and stored at -80°C.

### Real time quantitative PCR (RT-qPCR) of *HTT* and *Darpp-32*

Total RNA was isolated from R6/2 tissue samples after homogenization in MagNALyser (Roche, Basel, Switzerland) using MagNALyser green bead tubes (Roche) and RNA-Bee (50 mg tissue/ml RNA-Bee (Tel Test, Inc)) according to the manufacturer’s instructions, and subsequently treated with DNase I (DNA-free; ThermoFisher, Waltham, MA). Approximately 200 ng of total RNA was subjected to cDNA synthesis with random hexamers using the SuperScript II first-strand synthesis system (Invitrogen, Carlsbad, CA) in a total volume of 20 μl. Three μl of 1/40 cDNA dilution preparation was subsequently used in RT-qPCR analysis in presence of iQ^™^ SYBR^®^ Green Supermix (Bio-Rad). Standard RT-qPCR procedures were followed for the gene expression analysis of normalization genes and R6/2 m*HTT*, endogenous mouse *Htt* and *Darpp-32*. RT-qPCR primers for human *HTT* and *Darpp-32* were designed based on NCBI database sequence information and product identity was confirmed by DNA sequencing. All RT-qPCR reactions were run on the ViiA^™^ 7 Real-Time PCR System (Applied Biosystems, Foster City, CA) and included no template (NT) and no reverse transcriptase (RT-) controls. ViiA 7 RUO Software results were subjected to ΔΔCt analysis [41]. Obtained gene expression values from analysis of R6/2-derived samples were normalized to *Gapdh*, *Rab2* and *Ywhaz* expression. All samples were analyzed in three technical replicates. The sequence of used PCR primers is listed in S1 Table. For the cleanup procedure in S1 Fig transcript-bound (CUG)7 was removed prior to cDNA synthesis using a protocol adapted from Busan and Weeks [42]. In short, approximately 2 μg of RNA was diluted in 100 μl RNase free water, denatured at 95°C for 3 minutes and cooled on ice. After denaturation, samples were washed and eluted using RNeasy MinElute columns (Qiagen, Hilden, Germany). Two serial rounds of denaturation, washing and elution were performed prior tocDNA synthesis.

### TR-FRET analysis of mHTT protein levels

Time-resolved Förster resonance energy transfer (TR-FRET) immune-assay was used to quantify levels of both soluble and aggregated mHTT protein in R6/2 and soluble mHTT in Q175 (aggregated form assay is not optimized for Q175) [22]. TR-FRET analyses were performed at IRBM (Rome, Italy). The assay for soluble mHTT uses two labeled monoclonal antibodies (MW1 and 2B7) directed toward proximal N-terminal epitopes. One of the antibodies, MW1, is specific for the elongated polyQ stretch through which mHTT aggregates. The epitope is therefore masked in the presence of mHTT aggregates, resulting in loss of TR-FRET signal. In contrast, mHTT aggregates will present multiple binding sites for one single antibody in close proximity (antibody MW8). This allows simultaneous binding of the monoclonal antibody labeled with donor and acceptor fluorophores and thus generation of a TR-FRET signal. Brain tissue was homogenized in 1X lysis buffer (1X PBS, 0.4% Triton X and Protein Inhibitors) using ceramic beads containing vials and the Fastprep96 homogenizer (1600 rpm, 3 cycles, 30 sec/cycle). Homogenized samples were stored overnight at -80°C. The day after samples were reformatted in three 96-well plates and their concentration was determined using the BCA assay. TR-FRET assays were performed in 384-well plates to detect soluble HTT, using 2B7 and MW1 antibodies, and exon 1 HTT aggregates, using MW8 antibody [22].

Determination of the optimal protein amount to be analyzed was assessed on serial dilution curves of representative samples for each tissue, and was found to be 20–30 μg, depending on the brain region. This quantity was analyzed in both the TR-FRET assays for all the samples in three replicates. Assay plates were incubated at room temperature for 1 hour to detect the soluble form of the mHTT protein and 16 hours to detect aggregated mHTT. The fluorescence ratio (665/615) values were acquired by Envision (PerkinElmer) plate reader.

### Wes protein quantification

Protein quantification of mutant and endogenous HTT and of polyQ proteins HCN1, ATXN3, ATXN7 and TBP (Fig 7) was performed using a Wes^™^ protein analysis system (ProteinSimple, San Jose, CA, USA), following manufacturer’s instructions. Prior to quantification, optimal antibody dilution and total protein concentration were determined for each assay separately. Peak specificity was assessed by including no protein controls. HTT expression was assayed in the 66–440 kDa Wes Separation kit. Identity of endogenous and mutant HTT protein was verified by comparing signal of Q175 protein samples with those obtained from WT animals (Fig 7A). For other proteins containing a poly-Q stretch, the 12–230 kDa Wes Separation kit was used. In all cases, protein expression (area under the curve) was normalized for vinculin (VLC) signal. Details on the used antibodies, dilutions and amount of protein are listed in S2 Table.

### Statistical analysis

All values are presented as mean ± Standard Error of Mean (SEM), and differences were considered to be statistically significant at the P<0.05 level. Statistical analyses of all data were performed using t-test or One Way ANOVA (when appropriate) followed by posthoc testing (Graphpad prism Version 5.03).

## Supporting information

S1 Fig RT-qPCR amplification of m*HTT* and endogenous mouse *Htt* mRNA. (A) RT-qPCR detection of mutant *HTT* mRNA in cortex, relative to VEH-treatment and normalized for RNA input against averaged expression levels of *Ywhaz*, *Rab2* and *Gapdh*. On the top the location of the RT-qPCR primers in mutant *HTT* mRNA are indicated. RT-qPCR detection of m*HTT* mRNA is depicted before and after a cleanup step consisting of denaturation and centrifugation on a spin column (RNeasy MinElute columns, Qiagen) to remove (CUG)7 from the transcript. After this cleanup step the reduced RT-qPCR detection of m*HTT* mRNA is reversed. Data are presented as mean ± SEM (n = 14–15). Significance was assessed using One Way ANOVA followed by Dunnett’s multiple comparison posthoc test (*p<0.05, ***p<0.001 compared to R6/2 VEH). (B) RT-qPCR detection of endogenous mouse *Htt* mRNA in cortex, relative to VEH-treatment and normalized for RNA input against averaged expression levels of *Ywhaz*, *Rab2* and *Gapdh*. On the top the location of the RT-qPCR primers in endogenous *Htt* mRNA are indicated. For endogenous *Htt* mRNA with a short stretch of 4 CAGs no reduction of RT-qPCR was observed (no cleanup step applied). Data are presented as mean ± SEM (n = 14–15). No significant differences were observed using One Way ANOVA followed by Dunnett’s multiple comparison posthoc test. (C) RT-qPCR detection of mutant *HTT* mRNA in striatum and hippocampus, relative to VEH-treatment and normalized for RNA input against averaged expression levels of *Ywhaz*, *Rab2* and *Gapdh*. No cleanup procedure was applied, similar to the data in cortex before cleanup. Data are presented as mean ± SEM (n = 14–15). Significance was assessed using One Way ANOVA followed by Dunnett’s multiple comparison posthoc test (***p<0.001 compared to R6/2 VEH).
(TIF)

S2 Fig TR-FRET analysis of mHTT in cerebellum. Levels of soluble and aggregated mHTT protein in R6/2 cerebellum, as determined by TR-FRET-based immunoassay [22]. Each point is the average of 3 technical replicates. Data are presented as mean ± SEM. Significance was assessed using One Way ANOVA on R6/2 groups only followed by Dunnett’s multiple comparison posthoc test. Significance was not reached, but a trend was observed towards reduced mHTT protein levels in the high dose group in cerebellum for both soluble (p = 0.077) and aggregated (p = 0.066) mutant HTT protein.
(TIF)

S3 Fig PCA clusters and individual fine motor parameters of MotoRater data in R6/2 mice. (A) Principal component (PC) analysis of MotoRater data for pooled genders in R6/2 mice, showing the 10 PC’s contributing most of the variation, altogether 62.3% in the whole data. The percentage in each panel describes the proportion of the variation in the whole data set that each PC comprises. Data are presented as mean ± SEM. Significance was assessed using One Way ANOVA on R6/2 groups only followed by Dunnett’s multiple comparison posthoc test (*p<0.05 compared to R6/2 VEH). (B). Example of 5 individual fine motor parameters significantly affected by treatment with (CUG)7 in females and males. Data are presented as mean ± SEM. Significance was assessed using One Way ANOVA on R6/2 groups only followed by Dunnett’s multiple comparison posthoc test (*p<0.05, **p<0.01 compared to R6/2 VEH).
(TIF)

S1 Table Overview of qPCR primers and TaqMan assays. (DOCX)

S2 Table Overview of antibodies, antibody dilutions and amount of protein used for Wes analysis. (DOCX)

S1 Video Video clip of the motor performance of a VEH-treated female WT mouse from this study in the MotoRater setup. 9606_WT VEH: vehicle-treated female WT mouse in MotoRater. **The mice shown in the video clips are indicated in** Fig 4A **by circled data points.**
(MP4)

S2 Video Video clip of the motor performance of a VEH-treated female R6/2 mouse from this study in the MotoRater setup. 8028_R6.2 VEH: vehicle-treated female R6/2 mouse in MotoRater. ** mice shown in the video clips are indicated in** Fig 4A **by circled data points**.
(MP4)

S3 Video Video clip of the motor performance of a (CUG)7-treated female R6/2 mouse from this study in the MotoRater setup. 4585_R6.2 (CUG)7: (CUG)7-treated female R6/2 mouse in MotoRater. **The mice shown in the video clips are indicated in** Fig 4A **by circled data points.**
(MP4)
