# Supplementary figures and images for: The expanded CAG repeat in the huntingtin gene as target for therapeutic RNA modulation throughout the HD mouse brain
Source: PLoS One. 2017 Feb 9;12(2):e0171127. doi: 10.1371/journal.pone.0171127 (PMC5300196; doi:10.1371/journal.pone.0171127)

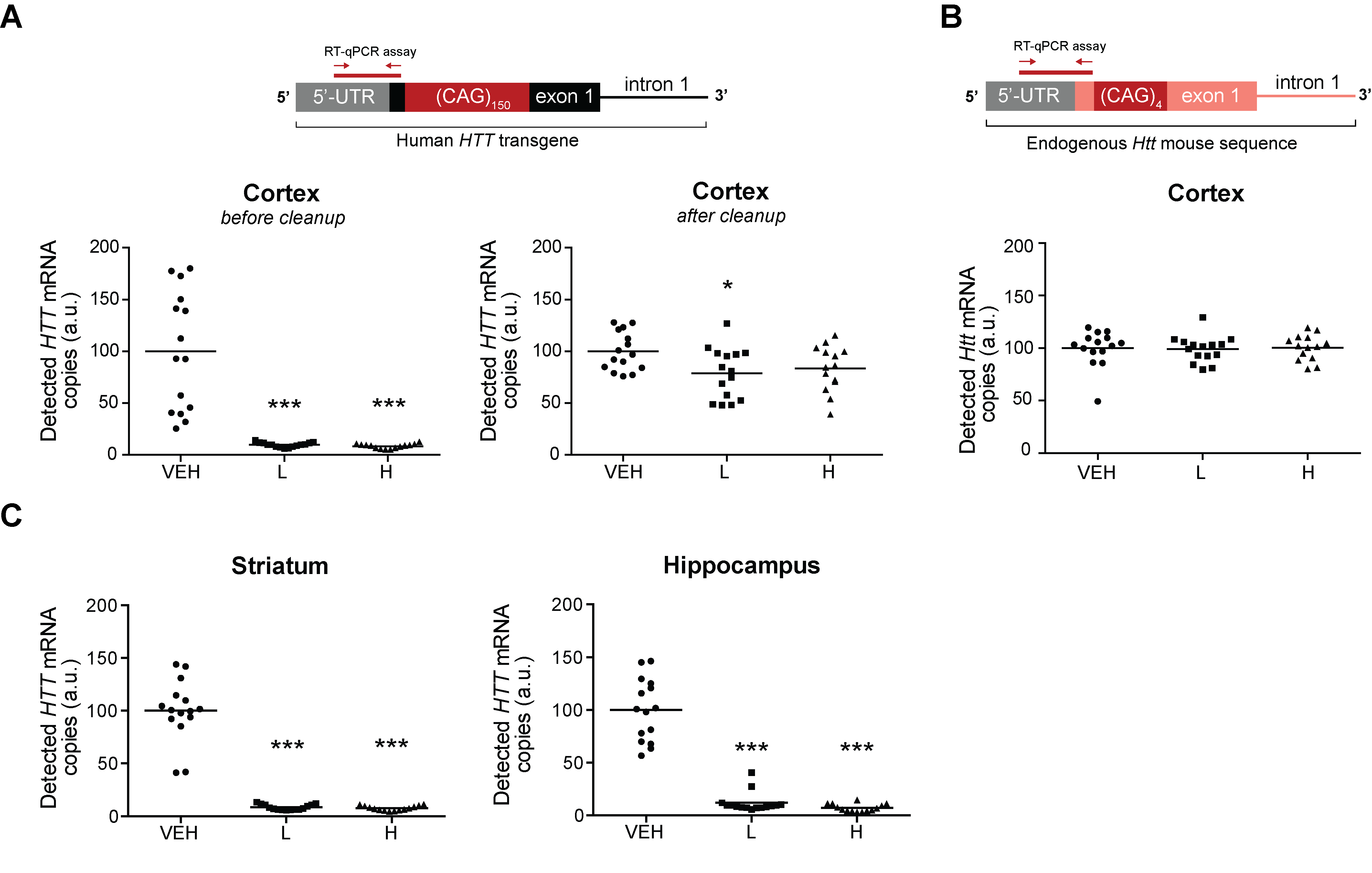

Supplement: S1 Fig — (A) RT-qPCR detection of mutant HTT mRNA in cortex, relative to VEH-treatment and normalized for RNA input against averaged expression levels of Ywhaz, Rab2 and Gapdh. On the top the location of the RT-qPCR primers in mutant HTT mRNA are indicated. RT-qPCR detection of mHTT mRNA is depicted before and after a cleanup step consisting of denaturation and centrifugation on a spin column (RNeasy MinElute columns, Qiagen) to remove (CUG)7 from the transcript. After this cleanup step the reduced RT-qPCR detection of mHTT mRNA is reversed. Data are presented as mean ± SEM (n = 14–15). Significance was assessed using One Way ANOVA followed by Dunnett’s multiple comparison posthoc test (*p<0.05, ***p<0.001 compared to R6/2 VEH). (B) RT-qPCR detection of endogenous mouse Htt mRNA in cortex, relative to VEH-treatment and normalized for RNA input against averaged expression levels of Ywhaz, Rab2 and Gapdh. On the top the location of the RT-qPCR primers in endogenous Htt mRNA are indicated. For endogenous Htt mRNA with a short stretch of 4 CAGs no reduction of RT-qPCR was observed (no cleanup step applied). Data are presented as mean ± SEM (n = 14–15). No significant differences were observed using One Way ANOVA followed by Dunnett’s multiple comparison posthoc test. (C) RT-qPCR detection of mutant HTT mRNA in striatum and hippocampus, relative to VEH-treatment and normalized for RNA input against averaged expression levels of Ywhaz, Rab2 and Gapdh. No cleanup procedure was applied, similar to the data in cortex before cleanup. Data are presented as mean ± SEM (n = 14–15). Significance was assessed using One Way ANOVA followed by Dunnett’s multiple comparison posthoc test (***p<0.001 compared to R6/2 VEH). (TIF) [file pone.0171127.s001.tif]

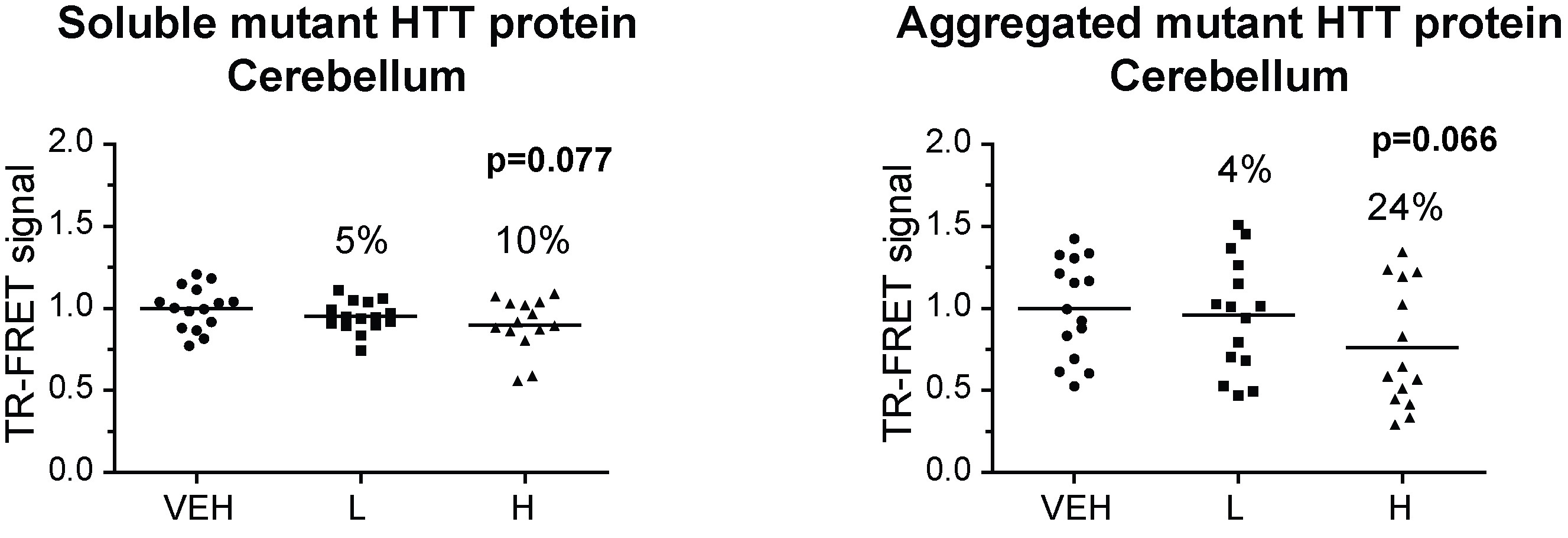

Supplement: S2 Fig — Levels of soluble and aggregated mHTT protein in R6/2 cerebellum, as determined by TR-FRET-based immunoassay [22]. Each point is the average of 3 technical replicates. Data are presented as mean ± SEM. Significance was assessed using One Way ANOVA on R6/2 groups only followed by Dunnett’s multiple comparison posthoc test. Significance was not reached, but a trend was observed towards reduced mHTT protein levels in the high dose group in cerebellum for both soluble (p = 0.077) and aggregated (p = 0.066) mutant HTT protein. (TIF) [file pone.0171127.s002.tif]

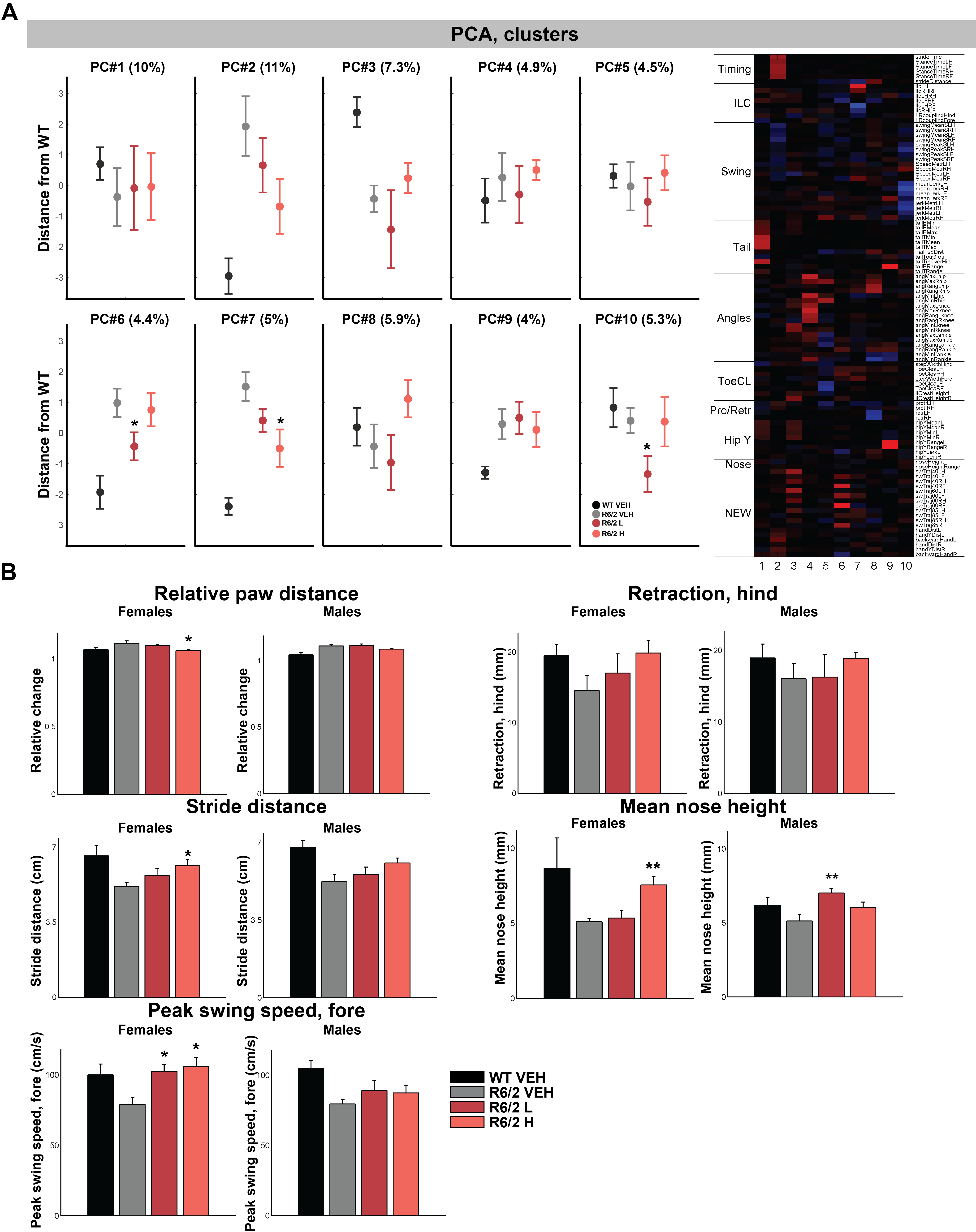

Supplement: S3 Fig — (A) Principal component (PC) analysis of MotoRater data for pooled genders in R6/2 mice, showing the 10 PC’s contributing most of the variation, altogether 62.3% in the whole data. The percentage in each panel describes the proportion of the variation in the whole data set that each PC comprises. Data are presented as mean ± SEM. Significance was assessed using One Way ANOVA on R6/2 groups only followed by Dunnett’s multiple comparison posthoc test (*p<0.05 compared to R6/2 VEH). (B). Example of 5 individual fine motor parameters significantly affected by treatment with (CUG)7 in females and males. Data are presented as mean ± SEM. Significance was assessed using One Way ANOVA on R6/2 groups only followed by Dunnett’s multiple comparison posthoc test (*p<0.05, **p<0.01 compared to R6/2 VEH). (TIF) [file pone.0171127.s003.tif]
