# Supplementary material for: The expanded CAG repeat in the huntingtin gene as target for therapeutic RNA modulation throughout the HD mouse brain
Source: PLoS One. 2017 Feb 9;12(2):e0171127. doi: 10.1371/journal.pone.0171127 (PMC5300196; doi:10.1371/journal.pone.0171127)
Supplement: S1 Table — (DOCX) [file pone.0171127.s004.docx]

|  |  |  |
| --- | --- | --- |
| **Gene** | **Primer name** | **Sequence** |
| Huntingtin, human (R6/2 mHTT transgene) | HTT-F | 5'-AGGTTCGCTTTTACCTGCGG-3' |
| Huntingtin, human (R6/2 mHTT transgene) | HTT-R | 5'-CATCAGCTTTTCCAGGGTCG-3' |
| Huntingtin, mouse (R6/2 endogenous *Htt)* | Htt-F | 5'-CCCCATTCATTGCCTTGCTG-3' |
| Huntingtin, mouse (R6/2 endogenous *Htt)* | Htt-R | 5'-CTTGAGCGACTCGAAAGCCT-3' |
| Dopamine and cAMP-regulated neuronal phosphoprotein 32, mouse | Darpp32-F | 5'- CCCAAAGTCGAAGAGACCCA-3' |
| Dopamine and cAMP-regulated neuronal phosphoprotein 32, mouse | Darpp32-R | 5'-CCGAAGCTCCCCTAACTCATC-3' |
| Ras-Related Protein Rab2, mouse | Rab2-F | 5'-TGGGAAACAGATAAAACTCCAGA-3' |
| Ras-Related Protein Rab2, mouse | Rab2-R | 5'-AATATGACCTTGTGATAGAACGAAAG-3' |
| Tyrosine 3-monooxygenase/tryptophan 5-monooxygenase activation protein, zeta, mouse | Ywhaz-F | 5'-AAATGAGCTGGTGCAGAAGG-3' |
| Tyrosine 3-monooxygenase/tryptophan 5-monooxygenase activation protein, zeta, mouse | Ywhaz-R | 5'-GGCTGCCATGTCATCGTAT-3' |
| Glyceraldehyde-3-phosphate dehydrogenase, mouse | Gapdh-F | 5'-GTCGGTGTGAACGGATTTG-3' |
| Glyceraldehyde-3-phosphate dehydrogenase, mouse | Gapdh-R | 5'-GAACATGTAGACCATGTAGTTG-3' |
