# Supplementary material for: The expanded CAG repeat in the huntingtin gene as target for therapeutic RNA modulation throughout the HD mouse brain
Source: PLoS One. 2017 Feb 9;12(2):e0171127. doi: 10.1371/journal.pone.0171127 (PMC5300196; doi:10.1371/journal.pone.0171127)
Supplement: S2 Table — (DOCX) [file pone.0171127.s005.docx]

| **Antibody (Ab)** | **Provider** | **Reference #** | **MW range** | **Ab dilution** | **Secondary Ab** | **Total protein** |
| --- | --- | --- | --- | --- | --- | --- |
| Anti-HTT | Atlas antibodies | HP-A026114 | 66-440 kDa | 1/20 | Anti-Rabbit (Wes) | 0.04 mg/ml |
| Anti-HCN1 | Abcam | ab176304 | 12-230 kDa | 1/10 | Anti-Rabbit (Wes) | 0.05 mg/ml |
| Anti-TBP | Abcam | ab125009 | 12-230 kDa | 1/10 | Anti-Rabbit (Wes) | 0.1 mg/ml |
| Anti-ATXN3 | Abcam | ab175265 | 12-230 kDa | 1/50 | Anti-Rabbit (Wes) | 0.1 mg/ml |
| Anti-ATXN7 | Abcam | ab11434 | 12-230 kDa | 1/10 | Anti-Rabbit (Wes) | 0.1 mg/ml |
| Anti-VCL | Cell Signaling | 13901S | 12-230 kDa | 1/500 | Anti-Rabbit (Wes) | 0.05 mg/ml |
